# Information coding by single spikes and bursts in thalamocortical relay neurons

**DOI:** 10.1186/1471-2202-12-S1-P367

**Published:** 2011-07-18

**Authors:** Fleur Zeldenrust, Pascal JP Chameau, Wytse J Wadman

**Affiliations:** 1 SILS-CNS, University of Amsterdam, Amsterdam, 1090 GE, the Netherlands

## 

The thalamus modulates the information flow to and from the cortex. The basal ganglia provide the thalamus with inhibitory input, whereas the cortex sends excitatory connections to the thalamus. Thalamocortical relay (TCR) neurons can fire both single spikes and bursts: the latter ones after a low threshold T-type calcium current is activated from a sufficiently hyperpolarized membrane potential. The question what these spikes and bursts code for and how the basal ganglia can influence this coding still remains open.

Information processing of TCR neurons was investigated in a computational model and compared with data recorded from TCR neurons in-vitro patched in rat thalamic brain slices. Gaussian noise superimposed on a DC current that put the neuron in different firing regimes (bursting or spiking) was injected into the TCR neuron and several computational tools to measure the reliability, precision and information content of resulting spike trains and coherence between input and output were used to analyze the recorded spike trains. Reverse correlation techniques (Spike-Triggered Average (STA) and Spike-Triggered Covariance (STC)) revealed what input features the neurons were most sensitive to in the different regimes.

The results of the analysis show that the two firing regimes of the TCR neurons, bursting mode and spiking mode, represent different coding strategies. At a low membrane potential, the neuron is in bursting mode, in which bursts phase-lock preferentially to low frequencies in the input signal, up to about 20 Hz, and single spikes show little reliability. The STA and filters of the STC analysis show that bursts are a response to long-lasting (up to 200 ms) input signals with a strong negative component. At more depolarized membrane potentials the neuron shows a gradual change from a bursting to a spiking regime. In this second regime single spikes are more precise and more reliable, earlier in time, and phase lock to input frequencies up to 60 Hz. The peaks of the STA become narrower, which reflects a shorter integration time and lower input resistance of the neuron. The STC filters show that spikes are more selective for fast fluctuations in this regime.

These results show that even at the single neuron level the thalamus can use different coding strategies depending on whether it is in a more hyperpolarized state, for instance as a consequence of strong basal ganglia input, or in a more depolarized state, induced by strong cortical input. This implies that single neurons can change their encoding regime depending on the background activity of the surrounding network.

